# Assessment of Anatomic Restoration of Distal Radius Fractures Among Older Adults: A Secondary Analysis of a Randomized Clinical Trial

**DOI:** 10.1001/jamanetworkopen.2019.19433

**Published:** 2020-01-17

**Authors:** Kevin C. Chung, Hoyune E. Cho, Yeonil Kim, H. Myra Kim, Melissa J. Shauver

**Affiliations:** 1 Section of Plastic Surgery, Department of Surgery, Michigan Medicine, Ann Arbor; 2 Section of Plastic Surgery, Department of Surgery, University of Michigan Medical School, Ann Arbor; 3 Early Development Statistics, Merck & Co Inc, Rahway, New Jersey; 4 Department of Biostatistics, University of Michigan, Ann Arbor

## Abstract

**Importance:**

The value of precise anatomic restoration for distal radius fractures (DRFs) in older adults has been debated for many decades, with conflicting results in the literature. In light of the growing population of adults aged 60 years and older, both fracture incidence and associated treatment costs are expected to increase.

**Objective:**

To determine the association between radiographic measures of reduction and patient outcomes after DRF in older patients.

**Design, Setting, and Participants:**

Data were collected from the Wrist and Radius Injury Surgical Trial (WRIST), a multicenter randomized clinical trial of DRF treatments for adults aged 60 years and older (enrollment from April 10, 2012, to December 31, 2016, with a 2-year follow-up). Data analysis was performed from January 3, 2019, to August 19, 2019. WRIST participants who completed 12-month assessments were included in the study. According to the biomechanical principle of alignment, 2-phase multivariable regression models were adopted to assess the association between radiographic measures of reduction and functional and patient-reported outcomes 12 months following treatment.

**Interventions:**

Participants were randomized to receive volar locking plate, percutaneous pinning, or external fixation. Those who opted for nonoperative treatment received casts.

**Main Outcomes and Measures:**

Hand grip strength, wrist arc of motion, radial deviation, ulnar deviation, the Michigan Hand Outcomes Questionnaire (MHQ) total score, MHQ function score, and MHQ activities of daily living score were measured at 12 months following treatment.

**Results:**

Data from 166 WRIST participants (144 [86.7%] women; mean [SD] age, 70.9 [8.9] years) found that only 2 of the 84 correlation coefficients calculated were statistically significant. For patients aged 70 years or older, every degree increase in radial inclination away from normal (22°) grip strength in the injured hand was 1.1 kg weaker than the uninjured hand (95% CI, 0.38-1.76; *P* = .004) and each millimeter increase toward normal (0 mm) in ulnar variance was associated with a 10.4-point improvement in MHQ ADL score (95% CI, −16.84 to −3.86; *P* = .003). However, neither of these radiographic parameters appeared to be associated with MHQ total or function scores.

**Conclusions and Relevance:**

The study results suggest that precise restoration of wrist anatomy is not associated with better patient outcomes for older adults with DRF 12 months following treatment. Surgeons can consider this evidence to improve quality of care by prioritizing patient preferences and efficient use of resources over achieving exact realignment.

**Trial Registration:**

ClinicalTrials.gov identifier: NCT01589692

## Key Points

**Question:**

What is the association between radiographic measures of reduction and patient outcomes 12 months after distal radius fractures treatment for adults aged 60 years or older?

**Findings:**

This secondary analysis of a multicenter randomized clinical trial on distal radius fractures treatment options included 166 patients who completed 12-month assessments. Radiographic parameters were not associated with functional and patient-reported outcomes.

**Meaning:**

Precise anatomic restoration does not guarantee good outcomes and may not have value in outcome evaluation for older adults with distal radius fractures.

## Teaser

This secondary analysis of a randomized clinical trial assesses the association between radiographic measures of reduction and functional and patient-reported outcomes after distal radius fracture in patients aged 60 years and older.

## Introduction

Nearly 90 000 older adults in the United States experience distal radius fractures (DRFs) annually.^1^ Accounting for nearly 20% of all fractures seen by physicians, DRF is the second most common type of fracture experienced by older adults.^2^ The functional impairment and disability from DRF in older adults can be long-lasting, with substantial consequences on independent living.^3^ There is concern for increased fracture incidence and escalating treatment costs as the population of older individuals increases and becomes more susceptible to falls and DRF. In 2005, the annual cost of treating DRF in the population aged 65 years and older was approximately $500 million.^4^ By 2025, it is estimated that annual fracture incidence and treatment costs will rise by 20%.^4^

Previous studies of DRF outcomes in persons who are aged 60 years and older report that precise anatomic reduction is not necessary to achieve satisfactory functional results because this population requires less functional recovery than younger patients.^5,6,7,8,9,10,11,12^ However, these conclusions cannot be applied to the current population of older adults who are much more active and functionally independent than previous generations.^13,14,15^ The perceived effect of disability from DRF will be more pronounced in the current population aged 60 years and older with greater demand in functional capacity. Studies have shown evidence that older adults place tremendous value on independent living, with reports of dependence making them feel as if they are a burden and delivering a toll on their emotional well-being.^3,16,17^ Some older adults even stated that they would accept increased mortality if it meant they would be functionally independent.^18^ Previous schools of thought on radiographic evaluation of reduction must be examined again with a more contemporary cohort of older patients.

What adds to the necessity of a renewed investigation is that many of the prior studies that found no clinically relevant differences in outcomes based on radiographic measures lacked rigor in sample size and study design.^19,20^ A systematic review of studies that evaluated radiographic measures of reduction after DRF found no consistency in the types of fracture or severity of DRF included in study cohorts, definitions or methods of obtaining various radiographic measures, or the acceptability criteria for malalignment.^21^ The substantial variability in the current literature demonstrates a need for a study that strives to establish a model of measuring and using radiographic variables to assess outcomes after DRF.^22,23^

In this study, we apply the rich and robust data collected from a large randomized clinical trial evaluating DRF treatment options to investigate the value of radiographic assessment in outcomes evaluation after DRF in older adults. Our results may help derive a definitive answer to the long-standing question of DRF management for contemporary populations of older adults and provide quality evidence for hand surgeons to tailor treatment plans to better fit patients’ needs. We hypothesized that radiographic measures of anatomic restoration are positively associated with both functional and patient-reported outcomes 12 months after DRF treatment for older adults.

## Methods

### Study Cohort

We used data collected as part of the Wrist and Radius Injury Surgical Trial (WRIST), an international, 24-site randomized clinical trial of DRF treatment in older adults.^24^ At each participating site, 2190 patients aged 60 years or older were screened for eligibility from April 10, 2012, to December 31, 2016. Inclusion criteria were isolated DRFs (exception: concomitant ulnar styloid fracture) with displacement warranting surgical intervention (AO [Arbeitsgemeinschaft für Osteosynthesefragen (Association for the Study of Internal Fixation)] type A2, A3, C1, or C2 and meeting one of the following radiographic criteria after reduction attempt: dorsal tilt >10°, radial inclination <15°, or radial shortening >3 mm). All fractures were amenable to treatment with all 3 surgical options. Patients with open fractures, bilateral fractures, prior ipsilateral DRF, or additional serious trauma were ineligible. Also excluded were patients living in nursing homes or other assisted living facilities and those with neurologic conditions affecting upper extremity sensation or movement, comorbid conditions prohibiting surgery, serious neurologic or psychiatric conditions precluding informed consent, or inability to complete study questionnaires and follow directions. The trial protocol is available in Supplement 1. The parent study WRIST was approved by the institutional review board at the University of Michigan, which included secondary analyses of the data.

Enrolled patients were randomized (after written consent was obtained) to receive percutaneous pinning, external fixation with or without supplemental k-wire fixation, or internal fixation with volar plate, stratified by study site. Those who consented to study participation but did not wish to undergo surgery despite the recommendation and who met identical eligibility criteria as randomized participants were treated with casting and were considered an observation group (eFigure 1 in Supplement 2). Follow-up care and rehabilitation and/or physical therapy were carried out per institutional standards at each site with 2 years of follow-up. For the present study, we included in our cohort the participants who completed 12-month functional evaluation, patient-reported outcomes questionnaires, and radiographic assessment.

### Variables of Interest

In our study, we included 12-month measurements of the clinical parameters recommended as standard components of outcomes assessment by the Distal Radius Outcomes Consortium, such as bilateral hand grip strength, arc of motion, patient-reported disability and functional outcomes as assessed with the Michigan Hand Outcomes Questionnaire (MHQ), and alignment as measured on plain radiographs.^25^ All objective functional outcomes were measured by trained study coordinators at each participating site of the WRIST. Grip strength was measured with a hydraulic hand dynamometer set to the second rung.^26^ Arc of motion, radial deviation, and ulnar deviation were measured with a goniometer. Patient-reported outcome variables included MHQ total score, as well as MHQ function and activities of daily living (ADL) domain scores.^27,28^ For radiographic evaluation of reduction, alignment was measured on plain radiographs obtained at 12-month assessment. All radiographs were stored in Digital Imaging and Communications in Medicine format, and Picture Archive and Communicating System imaging software was used to view and measure the radiographic parameters. We included radial inclination, radial height, ulnar variation, and tilt (dorsal angles were recorded as negative: eg, dorsal 10° = −10°) because those were the parameters with high interrater and intrarater agreement.^22^ These variables were measured by 2 trained clinical staff members, based on definitions by the American Academy of Orthopaedic Surgeons.^29^ All radiographs were measured twice, and those with discrepancies of more than 10% were resolved by a third reading.

Key clinical and demographic covariates included in our analysis model were sex, race, highest educational level, income, smoking status, preinjury level of activity, employment status 12 months following treatment, and status of dominant-hand injury. Race was self-reported by participants; this information was collected as required for all federally funded research projects. At enrollment, participants were asked to report their preinjury level of activity using the Rapid Assessment of Physical Activity, a 9-item questionnaire that evaluates a patient’s physical activity as sedentary, underactive, or active, based on guidelines from the Centers for Disease Control and Prevention.^30^

### Statistical Analysis

Our analytic goal was to assess the association between 12-month posttreatment radiographic measures of reduction and patient outcomes. We divided the cohort into 2 groups based on median age (70 years) to clearly portray the association in each age cohort. In addition to the level of function and patient-reported outcomes in the injured hand, we calculated the difference in measurements and scores between the injured hand and uninjured hand (eg, difference in grip strength as uninjured hand minus injured hand) to capture within-person recovery of function and patient-reported outcomes. Higher positive values would indicate less recovery achieved. Among the 4 radiographic parameters, we excluded radial height from our model because it was highly correlated with radial inclination (*r* = 0.94) (eFigure 2 in Supplement 2).

Based on the biomechanical principle of bony alignment, we hypothesized a U- or V-shaped association centered around the normal value for each radiographic parameter, at which point the difference in outcome between the injured and the uninjured hand is expected to be the smallest because we expected patients with perfect or near-perfect anatomic restoration to have better outcomes or greater recovery in function and MHQ scores than those with poor quality of reduction.^29^ This prognosis implies that the direction of association would differ for radiographic measures above and below the normal values; thus, we adopted a 2-phase multivariable regression model that permits separate regression coefficients above and below the normal values for each radiographic variable. We considered radial inclination of 22°, ulnar variance of 0 mm, and volar tilt of 11° as normal values, as defined by Taleisnik and Watson.^31^ Using the normal values as cutoffs, 2 slopes were calculated from the 2-phase multivariable regression model with all 3 radiographic variables included, adjusting for key covariates. For each radiographic variable, slope 1 assessed the association between the outcome Y and the values of the radiographic variable below or equal to the corresponding normal value adjusting for other radiographic values and covariates, and slope 2 examined the equivalent for values of the radiographic variable greater than the normal values. The regression equation for the 2-phase multivariable regression model is as follows:

Y = β_o_ + β_1_*X*_radinc_ + β_2_I(*X*_radinc_ >22) + β_12_(*X*_radinc_ × I[*X*_radinc_ >22])

+ γ_1_*X*_ulnavar_ + γ_2_I(*X*_ulnavar_ >0) + γ_12_{*X*_ulnavar_ × I(*X*_ulnavar_ >0)}

+ δ_1_*X*_tilt_ + δ_2_I(*X*_tilt_ >11) + δ_12_{*X*_tilt_ × I(*X*_tilt_ >11)}

+ η^T^*Z*_covariates_,

where

β_1_ = slope 1 when radial inclination ≤22;

β_1_ + β_12_ = slope 2 when radial inclination >22;

γ_1_ = slope 1 when ulnar variance ≤0;

γ_1_ + γ_12_ = slope 2 when ulnar variance >0;

δ_1_ = slope 1 when tilt ≤11;

δ_1_ + δ_12_ = slope 2 when tilt >11;

and β_12_, γ_12_, and δ_12_ = Δslope, for each respective radiographic variable.

Significantly different slopes (non-0 difference in slope [Δslope]) for each radiographic variable were considered consistent with the hypothesized association based on the biomechanical principle. We used a conservative level of α = .01 to be mindful of the multiple associations that we are assessing in our analysis. Statistical analyses were performed with R software version 3.5.3 (R Foundation).

## Results

### Sample Characteristics

The final study cohort included 166 patients (144 [86.7%] women; mean [SD] age, 70.9 [8.9] years) from the parent WRIST study who completed all 3 components of 12-month assessment: function tests, patient-reported outcome questionnaires, and radiographs. We included 89 participants aged 60 to 69 years in the younger group and 77 participants age 70 years or older in the older group. Demographic and clinical characteristics were generally similar between the 2 subgroups for sex, race, educational level, preinjury Rapid Assessment of Physical Activity category, and status of dominant-hand injury (Table 1). However, a greater proportion of participants in the younger group had income greater than or equal to $50 000 (49.4% vs 28.6%; *P* = .05). Likewise, there were more full-time workers in the younger group (16 [18.0%] vs 1 [1.3%]) and more retired participants in the older group (68 [88.3%] vs 52 [58.4%]) (*P* < .001). Among clinical characteristics, there were 10 active smokers in the younger group (11.2%) compared with 0 active smokers in the older group (*P* = .003) (Table 1). For radiographic measures, the mean (SD) for radial inclination was similar between the younger (21.4° [6.1°]) and older (19.8° [6.4°]) groups (*P* = .10). For ulnar variance, the mean (SD) displacement was less in the younger group (2.1 [2.0] mm) than the older group (3.1 [2.7] mm) (*P* = .01). The distribution of tilt measurements was also different between the younger (0° [11.6°] mm) and older (−5.2° [12.7°] mm) groups (*P* < .01).

**Table 1.** Study Cohort Characteristics by Age Group

Variable	No. (%)		*P* Value
	Younger, Aged 60-69 y	Older, Aged ≥70 y	
No. of patients	89	77	
Sex			
Female	78 (87.6)	66 (85.7)	.82
Male	11 (12.4)	11 (14.3)	
Race			
White	78 (87.6)	65 (84.4)	.49
Black	4 (4.5)	5 (6.5)	
Asian	3 (3.4)	5 (6.5)	
Other	4 (4.5)	1 (1.3)	
Missing	0	1 (1.3)	
Educational level			
<High school	23 (25.8)	30 (39.0)	.35
<Bachelor’s degree	7 (7.9)	4 (5.2)	
Bachelor’s degree	18 (20.2)	17 (22.1)	
≥Master’s degree	37 (41.6)	26 (33.8)	
Missing	4 (4.5)	0	
Income, $			
<10 000	2 (2.2)	6 (7.8)	.05
10 000-49 999	36 (40.5)	38 (49.4)	
50 000-69 999	18 (20.2)	9 (11.7)	
≥70 000	26 (29.2)	13 (16.9)	
Missing	7 (7.9)	11 (14.3)	
Smoking status			
Current	10 (11.2)	0	.003
Former	29 (32.6)	35 (45.5)	
Never	50 (56.2)	42 (54.5)	
Preinjury Rapid Assessment of Physical Activity			
Active	45 (50.6)	29 (37.7)	.18
Underactive	36 (40.4)	42 (54.5)	
Sedentary	7 (7.9)	6 (7.8)	
Missing	1 (1.1)	0	
Employment status at 12-mo			
Full-time	16 (18.0)	1 (1.3)	<.001
Part-time	10 (11.2)	6 (7.8)	
Retired	52 (58.4)	68 (88.3)	
Disabled or unemployed	11 (12.3)	2 (2.6)	
Dominant hand injured?			
Yes	44 (49.4)	32 (41.6)	.35
No	45 (50.6)	45 (58.4)	

### Two-Phase Model Results

Based on using a 2-phase multivariable regression model to describe the association between the radiographic measures of reduction and 12-month outcomes, we found that the Δslope was significant for only 3 models: (1) radial inclination vs difference in grip strength for the older group (Δslope = 1.00; 95% CI, 0.28-1.72; *P* = .01); (2) radial inclination vs difference in MHQ total score for the younger group (Δslope = −2.55; 95% CI, −4.45 to −0.64; *P* = .01); and (3) ulnar variance vs difference in MHQ ADL score for the older group (Δslope = 11.02; 95% CI, 4.57-17.47; *P* = .001) (Table 2).

**Table 2.** Testing the 2-Phase Model Fit for Association of Radial Inclination, Ulnar Variance, and Tilt With Patient Outcomes^a^

Outcome Variable by Age Group^b^	Radial Inclination		Ulnar Variance		Tilt	
	Slope Difference (95% CI)^c^	*P* Value	Slope Difference (95% CI)^c^	*P* Value	Slope Difference (95% CI)^c^	*P* Value
Grip strength difference						
Younger	−0.15 (−0.8 to 0.51)	.66	2.45 (−1.60 to 6.50)	.24	−0.13 (−1.18 to 0.93)	.82
Older	1.00 (0.28 to 1.72)	.01	1.08 (−1.78 to 3.94)	.46	0.33 (−0.54 to 1.21)	.46
Arc of motion difference						
Younger	−0.46 (−2.59 to 1.67)	.68	5.08 (−8.74 to 18.91)	.47	1.73 (−1.55 to 5.01)	.31
Older	3.12 (−0.17 to 6.41)	.07	−11.37 (−25.28 to 2.54)	.12	0.65 (−3.33 to 4.64)	.75
Radial deviation difference						
Younger	0.30 (−0.58 to 1.18)	.51	−0.26 (−5.98 to 5.47)	.93	−1.19 (−2.55 to 0.17)	.09
Older	−0.45 (−2.32 to 1.41)	.64	−0.003 (−7.40 to 7.39)	>.99	0.51 (−1.75 to 2.78)	.66
Ulnar deviation difference						
Younger	−0.35 (−1.25 to 0.56)	.46	1.65 (−4.29 to 7.58)	.59	−0.14 (−1.55 to 1.26)	.84
Older	0.85 (−1.04 to 2.74)	.38	−2.99 (−10.47 to 4.49)	.44	0.52 (−1.77 to 2.81)	.66
MHQ total score difference						
Younger	−2.55 (−4.45 to −0.64)	.01	11.34 (−1.07 to 23.75)	.08	2.02 (−0.92 to 4.97)	.18
Older	−1.21 (−3.91 to 1.49)	.38	−7.35 (−18.05 to 3.35)	.18	−0.70 (−3.98 to 2.58)	.68
MHQ function score difference						
Younger	1.05 (−1.17 to 3.27)	.36	5.01 (−9.47 to 19.50)	.50	−2.64 (−6.08 to 0.80)	.14
Older	2.93 (−0.95 to 6.81)	.15	11.12 (−4.24 to 26.47)	.16	0.35 (−4.35 to 5.05)	.88
MHQ ADL score difference						
Younger	−0.13 (−1.60 to 1.34)	.87	4.39 (−5.21 to 13.99)	.37	−2.01 (−4.32 to 0.24)	.08
Older	0.53 (−1.10 to 2.16)	.53	11.02 (4.57 to 17.47)	.001	0.61 (−1.37 to 2.58)	.55

Abbreviations: ADL, activities of daily living; MHQ, Michigan Hand Outcomes Questionnaire.

^a^ Covariates included in the model are sex, race, highest educational level, income, smoking status, preinjury level of activity, employment status 12 months following treatment, and status of dominant-hand injury.

^b^ Age group defined by median age (70 years): age 60 to 69 as the younger group and 70 years or older as the older group.

^c^ The slope difference is slope 1 minus slope 2, where slope 1 was calculated from data points with radial inclination less than or equal to 22°, ulnar variance less than or equal to 0 mm, or tilt less than or equal to 11°; and slope 2 from those with radial inclination greater than 22°, ulnar variance greater than 0 mm, or tilt greater than 11°.

From the three 2-phase models with appropriate fit, we calculated slope 1 and slope 2 for each model separately. For the older group, we found that the difference in grip strength between the injured and uninjured hands increased by 1.1 kg for each 1° increase in radial inclination above 22° (slope 2; 95% CI, 0.38-1.76; *P* = .004). In other words, for every degree increase in radial inclination away from normal, the injured hand’s grip strength was 1.1 kg weaker than the uninjured hand (Table 3 and Figure, A). We found that in the younger group, each 1° increase in radial inclination less than or equal to 22° was associated with a 1.3-point increase in MHQ total score difference between the injured and uninjured hands (slope 1: 95% CI, 0.28-2.35; *P* = .02) (Table 4 and Figure, B). This result contradicted our hypothesized association, as it indicates that for the younger group, each degree increase in radial inclination toward normal was associated with poorer recovery measured via MHQ total score. In addition, for the older group, each 1-mm increase in ulnar variance was associated with a 10.4-point decrease in MHQ ADL score difference when ulnar variance less than or equal to 0 mm (slope 1: 95% CI, −16.84 to −3.86; *P* = .003) (Table 4 and Figure, C). The negative slope indicates that each millimeter increase in ulnar variance toward the normal value of 0 mm was associated with a 10.4-point greater improvement in MHQ ADL score for the injured hand compared with the uninjured hand. We found similar results in terms of the direction of association by using the absolute 12-month outcome measurements in the injured hand as the response variable instead of the difference in outcomes between the injured and uninjured hands.

**Table 3.** Two-Phase Association Between Radiographic Measures and Functional Outcomes at 12 Months Following Treatment^a^

Outcome by Radiographic Variable by Age Group^b^	Slope 1 (95% CI)^c^	*P* Value	Slope 2 (95% CI)^c^	*P* Value
Grip strength difference				
Radial inclincation				
Younger	−0.07 (−0.40 to 0.26)	.69	−0.22 (−0.81 to 0.37)	.47
Older	0.07 (−0.19 to 0.33)	.61	1.07 (0.38 to 1.76)	.004
Ulnar variance				
Younger	−2.16 (−6.05 to 1.72)	.28	0.29 (−0.46 to 1.03)	.46
Older	−0.87 (−3.75 to 2.00)	.55	0.21 (−0.28 to 0.70)	.41
Tilt				
Younger	0.02 (−0.10 to 0.15)	.70	−0.10 (−1.15 to 0.95)	.85
Older	−0.11 (−0.20 to −0.01)	.03	0.23 (−0.65 to 1.10)	.61
Arc of motion difference				
Radial inclincation				
Younger	0.51 (−0.66 to 1.67)	.40	0.05 (−1.77 to 1.86)	.96
Older	−0.73 (−1.93 to 0.46)	.23	2.39 (−0.74 to 5.51)	.14
Ulnar variance				
Younger	−4.37 (−17.71 to 8.96)	.52	0.71 (−1.88 to 3.30)	.59
Older	12.74 (−1.25 to 26.74)	.08	1.37 (−0.88 to 3.62)	.24
Tilt				
Younger	−0.32 (−0.74 to 0.11)	.15	1.41 (−1.87 to 4.69)	.40
Older	0.04 (−0.40 to 0.48)	.88	0.69 (−3.28 to 4.66)	.74
Radial deviation difference				
Radial inclination				
Younger	0.24 (−0.24 to 0.71)	.33	0.54 (−0.22 to 1.29)	.17
Older	0.68 (0.00 to 1.36)	.06	0.23 (−1.55 to 2.01)	.80
Ulnar variance				
Younger	0.24 (−5.28 to 5.76)	.93	−0.02 (−1.09 to 1.05)	.97
Older	−0.52 (−7.95 to 6.92)	.89	−0.52 (−1.80 to 0.76)	.43
Tilt				
Younger	0.08 (−0.09 to 0.26)	.36	−1.11 (−2.46 to 0.25)	.12
Older	−0.15 (−0.40 to 0.10)	.25	0.36 (−1.89 to 2.62)	.75
Ulnar deviation difference				
Radial inclination				
Younger	0.18 (−0.31 to 0.68)	.47	−0.16 (−0.94 to 0.62)	.68
Older	−0.48 (−1.16 to 0.21)	.18	0.37 (−1.43 to 2.17)	.69
Ulnar variation				
Younger	−1.42 (−7.15 to 4.32)	.63	0.23 (−0.88 to 1.34)	.69
Older	3.53 (−3.99 to 11.05)	.36	0.55 (−0.75 to 1.84)	.41
Tilt				
Younger	−0.14 (−0.32 to 0.05)	.15	−0.28 (−0.69 to 1.12)	.70
Older	−0.01 (−0.27 to 0.24)	.91	0.51 (−1.77 to 2.79)	.66

^a^ Covariates included in the model are sex, race, highest educational level, income, smoking status, preinjury level of activity, employment status 12 months following treatment, and status of dominant-hand injury.

^b^ Age group defined by median age (70 years): age 60 to 69 years as the younger group and age 70 years or older as the older group.

^c^ Slope 1 was calculated from data points with radial inclination less than or equal to 22°, ulnar variance less than or equal to 0 mm, or tilt less than or equal to 11°; and slope 2 from those with radial inclination greater than 22°, ulnar variance greater than 0 mm, or tilt greater than 11°.

**Figure.**
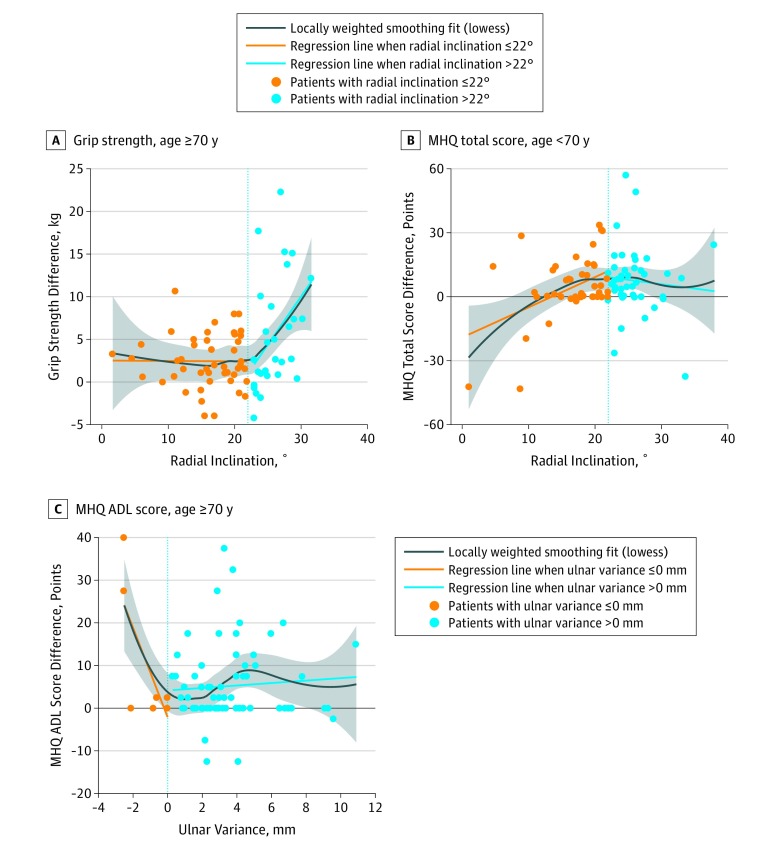
Multivariable 2-Phase Regression Plots The shaded area indicates the 95% CI. Vertical line indicates the normal value for each independent variable (eg, for panel A, the vertical line is at X = 22, which is the accepted normal value for radial inclination). ADL indicates activities of daily living; MHQ, Michigan Hand Outcomes Questionnaire.

**Table 4.** Two-Phase Association Between Radiographic Measures and Patient-Reported Outcomes at 12 Months Following Treatment^a^

Outcome by Radiographic Variable by Age Group^b^	Slope 1 (95% CI)^c^	*P* Value	Slope 2 (95% CI)^c^	*P* Value
MHQ total score				
Radial inclination difference				
Younger	1.31 (0.28 to 2.35)	.02	−1.23 (−2.86 to 0.40)	.14
Older	0.30 (−0.68 to 1.29)	.55	−0.91 (−3.48 to 1.66)	.49
Ulnar variance				
Younger	−10.51 (−22.49 to 1.46)	.09	0.83 (−1.49 to 3.15)	.49
Older	9.09 (−1.67 to 19.85)	.10	1.74 (−0.09 to 3.56)	.07
Tilt				
Younger	−0.04 (−0.42 to 0.34)	.85	1.98 (−0.96 to 4.93)	.19
Older	−0.12 (−0.48 to 0.24)	.53	−0.82 (−0.48 to 0.24)	.63
MHQ function score difference				
Radial inclination				
Younger	0.67 (−0.54 to 1.87)	.28	1.72 (−0.19 to 3.62)	.08
Older	0.20 (−1.21 to 1.61)	.78	3.13 (−0.56 to 6.82)	.10
Ulnar variance				
Younger	−5.65 (−19.62 to 8.32)	.43	−0.64 (−3.34 to 2.06)	.64
Older	−9.75 (−25.19 to 5.70)	.22	1.37 (−1.25 to 3.99)	.31
Tilt				
Younger	0.10 (−0.35 to 0.55)	.66	−2.54 (−5.97 to 0.89)	.15
Older	−0.12 (−0.63 to 0.40)	.66	0.24 (−4.45 to 4.92)	.92
MHQ ADL score difference				
Radial inclination				
Younger	0.83 (0.03 to 1.63)	.05	0.71 (−0.56 to 1.97)	.28
Older	0.42 (−0.17 to 1.02)	.17	0.95 (−0.60 to 2.50)	.24
Ulnar variance				
Younger	−3.56 (−12.82 to 5.71)	.45	0.83 (−0.96 to 2.63)	.37
Older	−10.35 (−16.84 to −3.86)	.003	0.67 (−0.43 to 1.77)	.24
Tilt				
Younger	0.10 (−0.20 to 0.39)	.53	−1.95 (−4.22 to 0.33)	.10
Older	−0.05 (−0.26 to 0.17)	.68	0.56 (−1.41 to 2.53)	.58

Abbreviations: ADL, activities of daily living; MHQ, the Michigan Hand Outcomes Questionnaire.

^a^ Covariates included in the model are sex, race, highest educational level, income, smoking status, preinjury level of activity, employment status 12 months following treatment, and status of dominant-hand injury.

^b^ Age group defined by median age (70 years): age 60 to 69 years as the younger group, and age 70 or older as the older group.

^c^ Slope 1 was calculated from data points with radial inclination less than or equal to 22°, ulnar variance less than or equal to 0 mm, or tilt less than or equal to 11°; and slope 2 from those with radial inclination greater than 22°, ulnar variance greater than 0 mm, or tilt greater than 11°.

On finding only a few associations with statistical significance, we also checked to see if the results changed substantively when unadjusted for other radiographic parameters, that is, only 1 radiographic variable was included in the regression model instead of all 3 variables. The results were similar, with the same 3 statistically significant associations with the same direction of association (eTable 1 and eTable 2 in Supplement 2).

## Discussion

In this study, we used data collected from an international, 24-site randomized clinical trial of DRF treatment options to investigate the value of precise anatomic restoration after DRF in older adults by examining the association between radiographic measures of reduction and outcomes 12 months following treatment. Our hypothesis was not confirmed; instead, radiographic measures were not associated with outcomes. In fact, only 3 of 14 models and 2 among 84 correlation coefficients were statistically significant. Furthermore, from the 3 regression models with appropriate fit, only 2 among 6 associations were congruent with the biomechanical principle of bony alignment: restoration of normal anatomic associations results in better function after DRF.^29^ Although we found that radial inclination greater than normal was associated with reduced grip strength relative to the uninjured hand and ulnar variance lower than normal was associated with lower MHQ score in adults aged 70 years and older, our results also indicate that these radiographic parameters were not associated with patients’ self-reported hand functional capacity (MHQ function scores) or overall hand health (MHQ total score). For adults aged 60 to 69 years, we found that radiographic measures of reduction did not appear to be associated with 12-month treatment outcomes.

Evidence in the literature does not agree on the role of radiographic alignment in DRF management for older adults. Some studies conclude that there is limited utility in precise fracture reduction in older adults,^5,6,10,11,19,32,33,34,35,36,37,38^ whereas others have found that the quality of anatomic reduction influences patient outcomes.^12,39,40,41,42,43,44,45,46,47^ For example, Arora et al^32^ conducted a randomized study of 75 patients aged 65 years and older with DRF and found that volar locking plate fixation produced better restoration of normal anatomy on plain films compared with nonoperative treatment, but radiographic measures were not correlated with any improvement in functional outcomes. In contrast, Brogren et al^46^ examined the Disabilities of the Arm, Shoulder, and Hand questionnaire scores at various levels of radiographic displacement for 123 patients and found that patients with severe displacement had worse Disabilities of the Arm, Shoulder, and Hand scores at 2 years. Moreover, even among the studies that found a significant association between radiographic variables and treatment outcomes, there is no consensus on which parameters are the most important or the magnitude of influence.^21^ The heterogeneity of conclusions on the applicability and predictive potential of radiographic measures of reduction, as noted in several large Cochrane Database meta-analyses, is most likely a result of wide variability in study cohorts and design: the severity of fracture, outcomes reported and the way they were measured, and the way radiographic parameters were named and defined.^48,49,50^

### Limitations and Strengths

There were some limitations to this study. Surgical technique, postoperative care, and therapy protocol care were not standardized in WRIST sites, and there may have been variations in care over the 24 participating sites and multiple surgeons. However, for our study, these differences increase the generalizability of our results by reflecting the variation in the real-world setting. The diversity in hospital settings and regional representation also adds to the robustness of the WRIST data. The parent WRIST study only included patients with substantially displaced DRF; thus, this is a limitation in our cohort selection. In addition, radiographic measurements on plain films are frequently shown to have low interrater and intrarater consistency. We minimized this bias by including only the radiographic parameters with a high rate of agreement,^22^ and discrepancies between the 2 sets of measurements were resolved with a third reading. We also measured radiographic variables according to the American Academy of Orthopaedic Surgeons’ definitions.^29^

The strengths of this study outweigh the limitations. We diminished the risk of selection bias and confounding by using the data collected from a multicenter randomized clinical trial. We included in our analysis the outcome measures recommended by the Distal Radius Outcomes Consortium,^25^ which promotes a standardized, unified approach for DRF outcomes assessment. Our use of a 2-phase multivariable regression model also adds rigor to our study as it reflects the biomechanical principle of bony alignment of the wrist more appropriately than single-phase regression models.

## Conclusions

The results of our study suggest that radiographic measures of reduction are not associated with functional outcomes and patient-reported outcomes in older adults after DRF treatment. Clinically, this finding implies that precise anatomic realignment of the wrist is not necessary for satisfactory outcomes. With this evidence, surgeons may elect to decrease operative time, use of resources, and associated costs that would have been spent to achieve perfect or near-perfect reduction. Decreasing time under anesthesia benefits patients as well with reduced risk of morbidity and mortality.^51^ In the treatment decision-making process, surgeons can prioritize patient preferences over the need to achieve exact realignment. Our study results may help to improve the quality of care in DRF management for older patients.

## Notes

Supplement 1. **Trial Protocol**

Supplement 2. **eFigure 1.** CONSORT Flow Diagram **eFigure 2.** Pairwise Correlation Matrix for Radiographic Variables **eTable 1.** Unadjusted Two-Phase Regression (Sensitivity Analysis) for Functional Outcomes at 12-Months Post-Treatment **eTable 2.** Unadjusted Two-Phase Regression (Sensitivity Analysis) for Patient-Reported Outcomes 12-Months Post-Treatment

Supplement 3. **Data Sharing Statement**
