# Supplementary material for: Assessment of Anatomic Restoration of Distal Radius Fractures Among Older Adults: A Secondary Analysis of a Randomized Clinical Trial
Source: JAMA Netw Open. 2020 Jan 17;3(1):e1919433. doi: 10.1001/jamanetworkopen.2019.19433 (PMC6991267; doi:10.1001/jamanetworkopen.2019.19433)
Supplement: Supplement 2. — eFigure 1. CONSORT Flow Diagram eFigure 2. Pairwise Correlation Matrix for Radiographic Variables eTable 1. Unadjusted Two-Phase Regression (Sensitivity Analysis) for Functional Outcomes at 12-Months Post-Treatment eTable 2. Unadjusted Two-Phase Regression (Sensitivity Analysis) for Patient-Reported Outcomes 12-Months Post-Treatment [file jamanetwopen-3-e1919433-s002.pdf]

Supplementary Online Content

Chung KC, Cho HE, Kim Y, Kim HM, Shauver MJ; WRIST Group. Assessment of anatomic restoration of distal radius fractures among older adults: a secondary analysis of a randomized clinical trial. *JAMA Netw Open*. 2020;3(1):e1919433. doi:10.1001/jamanetworkopen.2019.19433

eFigure 1. CONSORT Flow Diagram

eFigure 2. Pairwise Correlation Matrix for Radiographic Variables

eTable 1. Unadjusted Two-Phase Regression (Sensitivity Analysis) for Functional Outcomes at 12-Months Post-Treatment

eTable 2. Unadjusted Two-Phase Regression (Sensitivity Analysis) for Patient-Reported Outcomes 12-Months Post-Treatment

This supplementary material has been provided by the authors to give readers additional information about their work.

© 2020 Chung KC et al. *JAMA Network Open.*

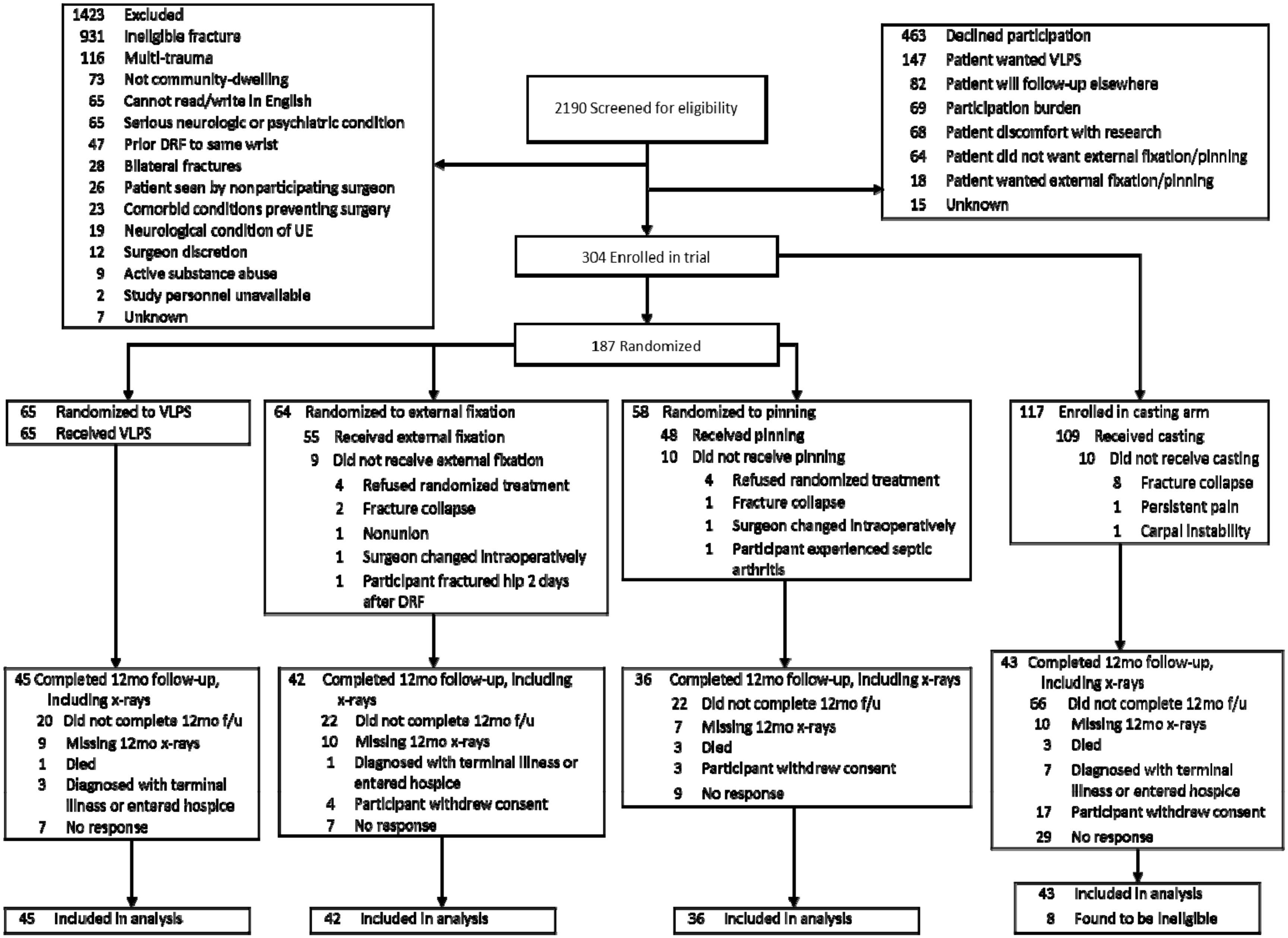

eFigure 1 CONSORT Flow Diagram

© 2020 Chung KC et al. *JAMA Network Open.*

eFigure 2. Pairwise Correlation Matrix for Radiographic Variables

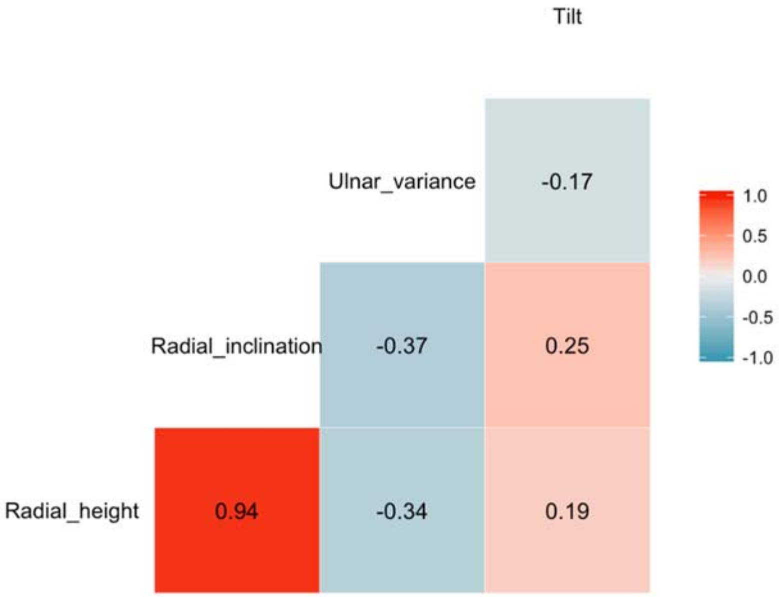

© 2020 Chung KC et al. *JAMA Network Open.*

eTable 1. Unadjusted Two-phase Regression (Sensitivity Analysis) for Functional Outcomes at 12-months post-treatment[a]

Outcome Variable	Radiographic Variable	Age Group	Slope 1	95% CL	P-value	Slope 2	95% CL	P-value
Grip Strength Difference	Radial Inclination	Younger	-0.14	-0.44, 0.17	0.39	-0.11	-0.59, 0.36	0.65
		Older	-0.02	-0.27, 0.23	0.88	1.09	0.47, 1.71	0.001
	Ulnar Variance	Younger	-1.45	-4.57, 1.67	0.37	0.26	-0.41, 0.92	0.46
		Older	-2.56	-5.32, 0.22	0.08	0.10	-0.41, 0.60	0.71
	Tilt	Younger	-0.009	-0.12, 0.10	0.87	-0.25	-1.21, 0.72	0.62
		Older	-0.05	-0.15, 0.04	0.29	0.24	-0.75, 1.23	0.63
Arc of Motion Difference	Radial Inclination	Younger	0.37	-0.73, 1.47	0.51	0.63	-0.96, 2.21	0.44
		Older	-0.78	-1.83, 0.28	0.16	1.00	-1.62, 3.62	0.46
	Ulnar Variance	Younger	-6.83	-18.97, 5.31	0.27	0.17	-2.40, 2.74	0.90
		Older	9.00	-3.38, 21.37	0.16	1.43	-0.66, 3.52	0.18
	Tilt	Younger	-0.13	-0.53, 0.26	0.51	1.91	-1.28, 5.09	0.24
		Older	-0.08	-0.46, 0.31	0.69	0.92	-2.97, 4.81	0.65
Radial Deviation Difference	Radial Inclination	Younger	0.32	-0.16, 0.79	0.19	0.48	-0.20, 1.17	0.17
		Older	0.52	-0.07, 1.11	0.09	0.36	-1.11, 1.83	0.63
	Ulnar Variance	Younger	-1.73	-6.61, 3.15	0.49	-0.07	-1.10, 0.96	0.90
		Older	-0.53	-7.00, 5.94	0.87	-0.83	-2.02, 0.36	0.18
	Tilt	Younger	0.09	-0.08, 0.25	0.31	-0.75	-2.06, 0.56	0.27
		Older	-0.03	-0.25, 0.19	0.81	0.38	-1.86, 2.62	0.74
Ulnar Deviation Difference	Radial Inclination	Younger	0.11	-0.35, 0.58	0.64	0.001	-0.68, 0.68	1.00
		Older	-0.52	-1.11, 0.08	0.09	0.13	-1.35, 1.61	0.86
	Ulnar Variance	Younger	-1.39	-6.25, 3.47	0.58	0.38	-0.66, 1.42	0.48
		Older	3.58	-2.90, 10.06	0.28	0.76	-0.43, 1.95	0.22
	Tilt	Younger	-0.14	-0.30, 0.02	0.08	-0.28	-1.55, 0.99	0.67
		Older	-0.08	-0.29, 0.14	0.48	0.54	-1.65, 2.72	0.63

[a] A separate regression model was run for each radiographic variable at a time. Slope 1 was calculated from data points with radial inclination $\leq 22^0$, ulnar variance ≤0mm, or tilt $\leq 11^0$; and slope 2 from those with radial inclination $>22^0$, ulnar variance >0mm, or tilt $>11^0$.

© 2020 Chung KC et al. *JAMA Network Open.*

eTable 2. Unadjusted Two-phase Regression (Sensitivity Analysis) for Patient-Reported Outcomes at 12-months post-treatment[a]

Outcome Variable	Radiographic Variable	Age Group	Slope 1	95% CL	P-value	Slope 2	95% CL	P-value
MHQ Total Score Difference	Radial Inclination	Younger	1.38	0.43, 2.33	0.006	-0.49	-1.87, 0.89	0.49
		Older	0.04	-0.85, 0.94	0.92	-2.3	-4.52, -0.08	0.05
	Ulnar Variance	Younger	-5.46	-16.15, 5.22	0.32	0.33	-1.93, 2.59	0.78
		Older	9.60	0.20, 19.00	0.05	1.26	-0.44, 2.97	0.15
	Tilt	Younger	0.02	-0.33, 0.37	0.92	1.48	1.34, 4.29	0.31
		Older	-0.13	-0.45, 0.20	0.45	-0.26	-3.55, 3.03	0.88
MHQ Function Score Difference	Radial Inclination	Younger	0.48	-0.66, 1.63	0.41	1.42	-0.24, 3.08	0.10
		Older	-0.15	-1.42, 1.12	0.81	3.17	0.01, 6.33	0.05
	Ulnar Variance	Younger	-11.35	-23.37, 0.66	0.07	-0.63	-3.16, 1.91	0.63
		Older	-13.51	-27.33, 0.31	0.06	0.81	-1.69, 3.32	0.53
	Tilt	Younger	0.07	-0.33, 0.48	0.72	-1.64	-4.90, 1.62	0.33
		Older	-0.09	-0.55, 0.38	0.71	0.45	-4.29, 5.19	0.85
MHQ ADL Score Difference	Radial Inclination	Younger	0.69	-0.06, 1.44	0.08	0.54	-0.55, 1.63	0.34
		Older	0.28	-0.32, 0.88	0.36	1.43	-0.07, 2.92	0.07
	Ulnar Variance	Younger	-6.96	-15.01, 1.08	0.09	0.72	-0.98, 2.42	0.41
		Older	-10.82	-17.06, -4.57	0.001	0.32	-0.81, 1.45	0.58
	Tilt	Younger	0.09	-0.18, 0.35	0.52	-1.48	-3.62, 0.67	0.18
		Older	0.005	-0.21, 0.22	0.96	0.70	-1.45, 2.85	0.53

© 2020 Chung KC et al. *JAMA Network Open.*

[a] A separate regression model was run for each radiographic variable at a time. Slope 1 was calculated from data points with radial inclination ≤22^{0}, ulnar variance ≤0mm, or tilt ≤11^{0}; and slope 2 from those with radial inclination >22^{0}, ulnar variance >0mm, or tilt >11^{0}.

© 2020 Chung KC et al. *JAMA Network Open.*
