# Supplementary material for: Assessment of Anatomic Restoration of Distal Radius Fractures Among Older Adults: A Secondary Analysis of a Randomized Clinical Trial
Source: JAMA Netw Open. 2020 Jan 17;3(1):e1919433. doi: 10.1001/jamanetworkopen.2019.19433 (PMC6991267; doi:10.1001/jamanetworkopen.2019.19433)
Supplement: Supplement 3. — Data Sharing Statement [file jamanetwopen-3-e1919433-s003.pdf]

Data Sharing Statement

Chung. Assessment of Anatomic Restoration of Distal Radius Fractures Among Older Adults. *JAMA Netw Open*. Published January 17, 2020. 10.1001/jamanetworkopen.2019.19433

Data
Data available: No

Additional Information
Explanation for why data not available: No data sharing planned at this time
